# Salt Consumption and Blood Pressure in Rural Hypertensive Participants: A Community Filed Trial

**DOI:** 10.1155/2022/2908811

**Published:** 2022-03-30

**Authors:** Farzaneh Noroozi, Mohammad Fararouei, Javad Kojuri, Leila Ghahremani, Kaveh Ghodrati

**Affiliations:** ^1^Department of Health Promotion, School of Health, Shiraz University of Medical Sciences, Shiraz, Iran; ^2^Department of Epidemiology, Shiraz University of Medical Sciences, Shiraz, Iran; ^3^Department of Cardiology, School of Medicine, Shiraz University of Medical Sciences, Shiraz, Iran; ^4^Research Center for Health Sciences, Institute of Health, Department of Health Promotion, School of Health, Shiraz University of Medical Sciences, Shiraz, Iran; ^5^Student Research Committee, Department of Health Promotion, School of Health, Shiraz University of Medical Sciences, Shiraz, Iran

## Abstract

**Purpose:**

Hypertension is a major cause of morbidity and mortality in the world. This study aimed to evaluate an intervention based on the Health Belief Model regarding the whole family's salt consumption and blood pressure among hypertensive patients in rural areas in Iran.

**Methods:**

This clinical multicenter trial (clinical and community) with a control and an intervention group was conducted on the residents of 14 villages covered by 14 health houses. Totally, 200 hypertensive patients (*n* = 100 in each group) were selected via multistage random sampling. The intervention included a two-day workshop on blood pressure and reducing salt consumption based on HBM structures for health personnel and an eight-session workshop on how to reduce salt intake and blood pressure for mothers who were responsible for the families' diets. Participants completed the questionnaires before and immediately after the intervention.

**Results:**

Compared to the control group, in the intervention group, a significant reduction was observed in salt consumption by the families (urine sodium and creatinine reduced by 35 mEq/l and 7.5 mg/dL, respectively). The results also revealed a significant decrease in blood pressure in the intervention group.

**Conclusion:**

The results showed that the mothers' model-based education could effectively improve the diet of the whole family members and, as a result, reduce the associated diseases. The main advantage of this study was the involvement of the rural health personnel, which helped run longer and larger-scale health-promotion programs in the communities.

## 1. Introduction

Hypertension is one of the most prevalent health issues among adults worldwide [1]. Hypertension is the major cause of direct and indirect morbidity and mortality, accounting for about 7.5 million deaths globally [2, 3]. Uncontrolled blood pressure is especially common in rural areas in low-and middle-income countries [4–6]. The prevalence of hypertension has been estimated to be 25–35% in Iran [7]. High blood pressure and its complications have been reported to be associated with individuals' lifestyles, particularly diet [8]. High salt intake has been reported as a major risk factor for high blood pressure [9–11]. Worldwide, 70 million disability-adjusted life-years and 3 million deaths in 2017 were attributed to high salt intake, making it one of the top 3 dietary risk factors [12, 13]. As a result, reducing salt intake was recommended to control the blood pressure and to reduce the risk of its complications [14]. The results of a study in Iran showed that approximately 79% of people with high blood pressure in a rural community add salt to their food when cooking and eating [15]. Many patients with high blood pressure do not pay enough attention to regular physical activity, a low-salt and high-fruit diet, and weight control [16, 17]. Education of health behaviors leads to prevention of exacerbation of signs and symptoms of the disease, reduction of mortality, and improvement of lifestyle [18]. These programs are on a very wide range of social and cultural bases and are concordant with different levels of knowledge and attitudes towards health issues, especially in the rural society of Iran. In this regard, cost-effectiveness, social and cultural adaptation, and sustainability of the programs are of great importance to reduce a wide variety of complications [19]. Studies have shown that interventions based on health education and community-based models in patients with hypertension in rural areas increased the average self-care behavior and decreased blood pressure in individuals [15, 20]. It is well accepted that people's attitudes or beliefs about a disease or a health behavior affect the acceptance of health behavior. The health belief model (HBM), a well-established and widely used psychological theory, explain how health beliefs influence peoples' health behaviors and why some people adopt certain health behavior, while others fail [21]. According to this model, people are encouraged to follow a healthy lifestyle whenever they find themselves at risk of diseases. The model measures the perceived susceptibility, severity of the disease, benefits of the healthy behavior, costs paid for the healthy behavior, guide to action, and changes in the factors that influence the behavior [14]. Many people with high blood pressure do not pay attention to proper diet as part of their treatment [16]. It seems that the root of these behaviors and misconceptions may be in patients' poor information and awareness of the nature of the disease and related nutrition, so that one of the reasons for not controlling hypertension is poor patient awareness [22]. This lack of accurate information has been reported as a worrying matter in rural areas [23]. Therefore, there is a need to increase educational interventions to increase knowledge and attitude in these areas. In community interventions, one of the ways to reduce inequalities related to health literacy is education through nonspecialist health educators and community health workers (CHWs) [24]. CHWs are people who are responsible for educating and providing services in rural areas [25]. In terms of ethnicity, language, socioeconomic status, and life experiences, CHWs are very similar to the members of the community they serve and also have a great ability to provide culturally appropriate health education to the people of their area [26]. Considering the effectiveness of educational models in promoting behaviors related to blood pressure control and the importance of the role of CHWs in education and improving the level of knowledge, attitude, and behavior of individuals [20, 27], a study aiming to examine the effect of an educational program based on health belief model by CHWs was performed on blood pressure of patients with hypertension in Sarvestan city, Fars province.

## 2. Materials and Methods

### 2.1. Study Design

**Figure 1:**
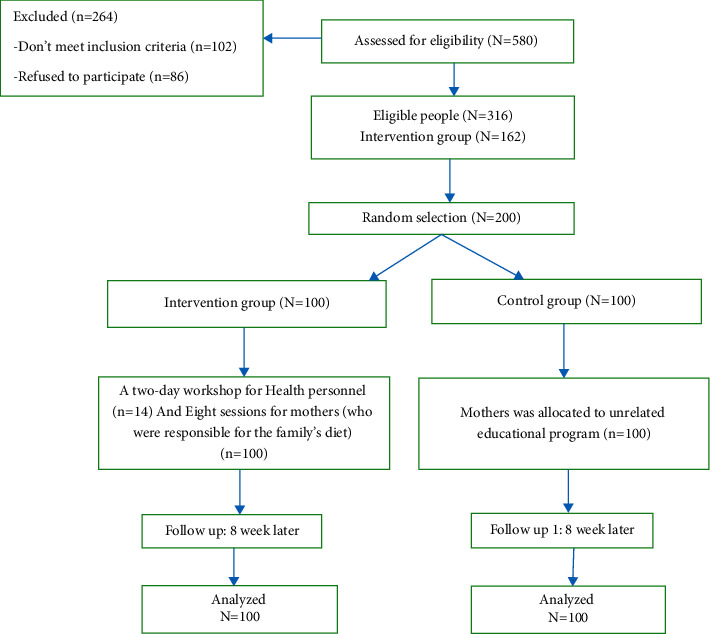
Consort flow chart of participants.

This randomized controlled field trial aimed to evaluate the impact of an educational program to reduce blood presser and salt intake among the residents of the study area. In Iran, primary healthcare including vaccination, family planning, and primary and general medical services are provided free of charge or at a low cost by the government for all rural and urban residents. Hence, all Iranian citizens are registered in and covered by this primary healthcare network, especially for vaccination, child growth, development monitoring, and maternal health services. In rural areas, these services are provided by health houses run by one or two health volunteers. Each health house covers a precisely defined population. Each member of the population and his/her family members are registered in a health house, and their data are recorded in a file called the “family file.” Furthermore, a hypertension screening program is routinely conducted for all rural and urban populations. In the present study, a sample of hypertensive patients was selected (not more than one patient from each family) and followed for monitoring urine sodium and creatinine concentrations as well as blood pressure. High blood pressure was defined as a systolic blood pressure higher than or equal to 140 mmHg and a diastolic blood pressure higher than or equal to 90 mmHg. It should be noted that the study population and patients were the residents of Sarvestan, a rural area in Fars province in 2017. Considering the power of 80% and error of 5%, the participants were selected through multistage cluster random sampling. In doing so, the rural health houses (*n* = 14) were first randomly divided into seven interventions and seven control areas irrespective of their geographic locations. Next, according to the list of hypertensive patients and proportion of the population covered by each health house, 100 patients were randomly selected from the health houses in the intervention group, and 100 ones were randomly selected from those in the control group (see Figure 1). The inclusion criteria of the study were being hypertensive with no other known underlying diseases, aging over 30 years, consuming family dishes (no particular diet), and living in Sarvestan. The exclusion criteria were being absent in more than one educational session, not having migrated to Sarvestan, and not having kidney disease.

### 2.2. Interventions

Firstly, the patients' family profiles (characteristics of the patients' first-degree relatives) were defined. Secondly, a two-day workshop was held. The HBM constructs and salt reduction program were explained for 12 CHWs in 7 health houses of the intervention group in the first and second sessions, respectively. In addition, eight sessions were held for mothers (who were responsible for the families' diets) in terms of diet and harms of consuming a high amount of salt. In this way, the CHWs from the intervention area attended a two-day workshop on the effects of salt on blood pressure and the circulatory system. The CHWs were then asked to deliver the information to mothers via a course containing eight sessions (one session per week) based on the HBM constructs (see Table 1). Patients with limited health literacy often rely only on oral instructions. Therefore, teaching should be interactive and nonspecialized and with clear verbal communication. In addition, the use of reinforcement techniques such as the use of photographs and shapes is suggested [24]. For education, group discussion and question and answer methods were used in addition to lectures, because the purpose of group learning techniques is to encourage and stimulate group discussions in a completely interactive way. Group discussion can increase knowledge and attitudes and change behavior in patients with hypertension by providing conditions such as expressing experiences and gaining information, challenging beliefs and attitudes, promoting motivation to change lifestyles, and increasing self-management [28]. In both control and intervention groups, routine educations were provided by health workers, but in the intervention group, in addition to routine educations, a program to reduce blood pressure and salt consumption was also implemented. On the other hand, in the control group, parenting styles education was provided to mothers.

The 24-hour urinary excretion was measured before and after the intervention. Blood pressure monitoring was also done routinely according to the national hypertension screening program irrespective of the patients' locations. Moreover, a researcher-made questionnaire based on the HBM was designed and applied to obtain information regarding the participants' salt consumption before and after the eight-week intervention.

### 2.3. Data Collection

The questionnaire was used to collect the data based on the HBM via a Likert scale. To determine the face and content validity of the questionnaire, 30 hypertensive patients were requested to fill it out. Then, the questionnaire was given to 10 experts. Based on the qualitative content validity and according to the recommendations of the experts as well as the ambiguities raised by the patients, changes were applied to the questionnaire items. Mean CVR and CVI values were 0.93 and 0.9, respectively.

Afterwards, the reliability of the questionnaire was assessed using Cronbach's alpha. The components of the HBM included perceived susceptibility (seven questions, total score ranging from 7 to 35, Cronbach's alpha = 0.71), perceived severity (five questions, total score ranging from 5 to 25, Cronbach's alpha = 0.91), perceived benefits (seven questions, total score ranging from 7 to 35, Cronbach's alpha = 0.88), perceived barriers (13 questions, total score ranging from 13 to 65, Cronbach's alpha = 0.79), and self-efficacy (11 question, total score ranging from 11 to 55, Cronbach's alpha = .076). The total Cronbach's alpha questionnaire was 0.81.

Blood pressure was measured twice with a 10-minute interval using a Mercury sphygmomanometer (ALP K2 Brand of V300) on the patients' right arms while they were sitting. The two measurements were performed in the morning, and the average blood pressure was recorded. Moreover, a 24-hour urinary excretion (measured via flame photometry) was measured in order to determine the urine creatinine level (mg/dL) and sodium level (mEq/l). The sodium and creatinine levels were measured in the laboratory of Shohada Hospital in Sarvestan. Additionally, height (cm) and weight (kg) were measured in the health centers using a measuring tape and a digital scale, respectively. All the centers in both intervention and control groups used similar instruments, which were calibrated at the opening hour of each working day. The 24-hour urine collection was done from 7 AM to 7 AM on the next day, and the patients were instructed to collect their urine samples even at night. After eight weeks (after educational sessions), participants in two groups (control and intervention) were again asked to go to the health houses, and blood pressure, weight, 24-hour urinary sodium, and HBM constructs were measured in two groups under the same conditions. After obtaining the ethical approval of the Ethics Committee of Shiraz University of Medical Sciences, the participants' written informed consents were gathered.

### 2.4. Statistical Analysis

The data were analyzed using the SPSS software (version 16). Paired sample *t*-test and independent sample *t*-test were applied, and the significance level was set at 0.05.

## 3. Results

All the 200 hypertensive patients were over 30 years of age, with the mean age of 60.92 ± 12.05 years. The patients had similar dishes shared with their families (no special diets) before and after the intervention. The demographic characteristics of the study participants have been presented in Table 2. Accordingly, the patients were predominantly females (77.5%) and homemakers (77%). No significant difference was found between the study groups regarding the demographic variables (*p* > 0.05).

As shown in Table 3, the mean scores of the HBM constructs were significantly different in the treatment and control groups eight weeks after the intervention. Based on the results of paired sample *t*-test, the mean scores of the constructs increased in the treatment group after the intervention (*p* < 0.05). However, the changes in the mean scores of the constructs were not significant among the control group participants (*p* > 0.05).

The results of paired sample *t*-test regarding the impact of education in the control and treatment groups have been shown in Table 4. Accordingly, the intervention had a significant effect on the reduction of systolic and diastolic blood pressure (means of reduction = 2.29 and 1.04 mmHg, respectively), urine sodium, creatinine, and salt consumption in the treatment group (*p* < 0.05).

## 4. Discussion

This study was performed to evaluate salt consumption and blood pressure in rural hypertensive participants, a community filed trial. The results of the present study showed that HBM-based educational intervention significantly improved the mean scores of HBM variables (perceived sensitivity, perceived severity, perceived benefits, perceived barriers, and self-efficacy) in patients. This results were consistent with those of other studies in the field [29–31]. Still, they were inconsistent with Ştefănuţ and Vintilă [32] study, in which HBM-based educational intervention did not make significant change in the mean scores in perceived susceptibility, self-efficacy, and barriers. The difference between the results of Stefano's study and the present study can be due to the average young age of the participants, the good health condition reported by most of them, and the lack of a history of breast cancer in the family for most of them. It can explain the fact that the participants did not perceive susceptibility against this disease. But in our study, the participants were middle-aged patients who understood the severity of the risk of hypertension and the severity of its complications in various physical, psychological, social, and economic dimensions and benefited from education to overcome obstacles and increased self-efficacy to improve their disease.

According to the results, the mothers' education program effectively reduced salt consumption and the patients' blood pressure. However, no significant changes were observed in control group. In some studies, educational intervention based on HBM has led to a reduction in patients' blood pressure, which is in line with our study [31, 33]. But in the Ramezankhani et al. study, education did not reduce diastolic blood pressure [34], which could be due to differences in the type of research community, reducing the sample size and the number of educational sessions. It is because in the mentioned study, 3 educational sessions were performed on 39 elderly people. The results of a number of studies, in line with our study, showed that education has been effective in reducing salt consumption [35, 36], But Manios's et al. intervention based on HBM did not reduce salt intake in postmenopausal women [37], which can be due to the long time between education sessions (once every two weeks) compared to weekly education sessions in our study. On the other hand, in our study, education was conducted on mothers who play a very important role in family nutrition style, so mothers' awareness of proper eating behaviors has a direct impact on family nutritional behavior [38]. In addition to awareness, education has increased their self-efficacy in reducing salt consumption, and since self-efficacy is a powerful mediator to improve healthy behaviors, changes in self-efficacy can result in positive lifestyle changes [39].

The results of the current study showed that the CHWs could be involved in simple but effective community-based health programs to reduce the communities' health problems. This was in line with the results of other interventional studies emphasizing the participation of community health personnel in dealing with different health issues including managing hypertension [20, 27], tuberculosis care [40], and Diabetes control [41]. Thus, by conducting training courses, CHWs who are predominantly well respected can act as role models in the community.

### 4.1. Strengths and Limitations

Despite many interventions in reducing blood pressure and salt consumption, one of the strengths of this study is performing a community-based intervention in a relatively large number of patients living in rural areas.

There were limitations in this study. The first was the short time interval between intervention and follow-up of samples, and the second was that the study was performed on patients covered by rural health centers, so it is not possible to generalize the results to urban areas.

## 5. Conclusion

The reduction in blood pressure in the intervention group in this study may have occurred because the development of health beliefs can help patients pay more attention to blood pressure [31]. On the other hand, group discussions and question-and-answer sessions gave patients the opportunity to talk about their current situation and the problems they have recently encountered. CHWs' responses to their doubts may have helped patients follow their recommendations [42].

The results of this field trial suggested that simple but well-designed educational programs by CHWs could promote the healthy behaviors of families so effectively. Using theory-based models, health personnel can act as good facilitators who can help communities get involved in recognizing and improving their health issues, particularly in rural areas.

## Figures and Tables

**Table 1:** Educational session's table.

Educational session number	Educational content	Educational method	Educational media	Duration of education
1	(i) Introducing the program goals	(i) Lecture	(i) Power Point	1/5 hour
	(ii) Learn more about hypertension and its causes	(ii) Question and answer	(ii) Educational clip	
			(iii) Picture	

2	(i) Explain the ways of prevention and nonpharmacological treatment of hypotension	(i) Lecture	(i) Power Point	2 hours
		(ii) Question and answer	(ii) Educational clip	
		(iii) Say positive experiences	(iii) Picture	

3	(i) The role of salt in increasing blood pressure	(i) Lecture	(i) Power Point	1/5 hour
	(ii) Recognize food labels	(ii) Question and answer	(ii) Educational clip	
	(iii) Hidden salts in food		(iii) Picture	
	(iv) Permitted daily consumption of salt and its consumption in Iran			

4	Create perceived sensitivity by expressing	(i) Lecture	(i) Power Point	2 hours
	(i) Prevalence of hypertension and its mortality in the world and in Iran	(ii) Question and answer	(ii) Educational clip	
	(ii) The role of family history in increasing blood pressure	(iii) Collaborative learning techniques	(iii) Picture	
	(iii) The effect of salt abuse on the incidence of diseases, especially blood pressure			

5	Create perceived intensity by expressing	(i) Lecture	(i) Power Point	1/5 hour
	(i) Complications and mortality of hypertension in all age groups	(ii) Question and answer	(ii) Educational clip	
	(ii) Side effects of salt consumption in all age groups	(iii) Collaborative learning techniques	(iii) Picture	

6	Create perceived benefits by expressing	(i) Group discussion	(i) Power Point	2 hours
	(i) The benefits and consequences of a healthy diet on reducing diseases, especially high blood pressure	(ii) Question and answer	(ii) Educational clip	
	(ii) Positive consequences of reducing salt consumption on reducing diseases, especially blood pressure	(iii) Collaborative learning techniques	(iii) Picture	
		(iv) Say positive experiences		

7	Reduce perceived barriers by expressing	(i) group discussion	(i) Power Point	2 hours
	(i) Barriers to eating a healthy diet and reducing salt intake	(ii) Question and answer	(ii) Educational clip	
	(ii) Training ways to overcome the temptation to consume unhealthy foods and salt	(iii) Collaborative learning techniques Say positive experiences	(iii) Picture	

8	Create perceived self-efficacy by expression	Group discussion question and answer Collaborative learning techniques Say positive experiences	(i) Power Point	2 hours
	(i) Ways to increase self-efficacy and its role in doing things		(ii) Educational clip	
	(ii) Training to prepare a healthy food by removing excess salt from food		(iii) Picture	
	(iii) Training alternative methods of salt and natural flavors in preparing daily food			
	(iv) Training alternatives to snacks and salty foods among family snacks			

**Table 2:** Demographic characteristics of the participants (*N* = 200).

Variable		Treatment (*N* = 100)		Control (*N* = 100)		*p* value
		Mean ± SD	*N* (%)	Mean ± SD	*N* (%)	
Age (Y)		59.78 ± 11.24		62.06 ± 12.86		0.18
Sex	Female		80 (80%)		75 (75%)	0.4
	Male		20 (20%)		25 (25%)	
Marital status	Single		4 (4%)		2 (2%)	0.34
	Married		96 (96%)		98 (98%)	
Weight (kg)		68.03 ± 14.07		66.28 ± 17.14	—	0.43
Height (cm)		159.91 ± 6.80		158.05 ± 8.31	—	0.08
Educational level; No. (%)	Elementary		94 (94%)		96 (96%)	0.37
	Higher		6 (6%)		4 (4%)	
Income, Riyal; No. (%)	Low (<1000000)		98 (98%)		99 (99%)	1.00
	Moderate (≥1000000)		2 (2%)		1 (1%)	
Job	Housewife		81 (81%)		73 (73%)	0.12
	Farmer		19 (19%)		27 (27%)	

**Table 3:** The score of health belief model components before and 8 weeks after the intervention in the treatment and control groups.

Health model constructs	Test time	Treatment group	Control group	*p* value
		Mean ± SD	Mean ± SD	
Perceived susceptibility	Before intervention	13.67 ± 1.79	13.38 ± 1.51	0.21
	After intervention	16.93 ± 2.04	13.21 ± 1.83	0.001
*p* value^		0.001	0.35	
Perceived severity	Before intervention	10.36 ± 4.10	10.49 ± 2.50	0.78
	After intervention	13.79 ± 3.94	10.71 ± 2.72	0.001
*p* value^		0.001	0.26	
Perceived benefits	Before intervention	12.32 ± 2.39	12.93 ± 2.59	0.08
	After intervention	15.22 ± 3.26	13.22 ± 2.75	0.001
*p* value^	—	0.001	0.29	—
Perceived barriers	Before intervention	38.75 ± 5.17	34.52 ± 2.43	0.08
	After intervention	35.60 ± 3.20	34.41 ± 2.95	0.007
*P* value^		0.001	0.63	
Self-efficacy	Before intervention	23.65 ± 3.76	23.26 ± 2.12	0.36
	After intervention	24.81 ± 3.28	23.35 ± 2.21	0.001
*p* value		0.002	0.73	

Significant *p* < 0.05.

**Table 4:** The effect of 8-week educational intervention on blood pressure and salt consumption.

Variables	Intervention group		Dif^*∗*^	*p* value	Control group		Dif^*∗*^	*p* value
	Before (SD)	After (SD)			Before (SD)	After (SD)		
Systolic blood pressure (mm Hg)	136.15 ± 13.74	133.86 ± 14.84	−2.29	0.02	136.16 ± 16.78	136.10 ± 16.64	0.95	0.90
Diastolic blood pressure (mm Hg)	81.46 ± 8.95	80.60 ± 8.29	−1.04	0.03	81.20 ± 10.06	81.25 ± 10.05	0.05	0.75
Urine sodium level (mEq/l)	140.26 ± 51.25	128.99 ± 52.17	−35.68	0.04	144.07 ± 32.39	142.59 ± 37.56	−3.85	0.73
Creatinine (mg/dL)	99.28 ± 45.25	87 ± 40.16	7.49	0.01	99.23 ± 45.34	96.49 ± 47.13	−3.48	0.66
Salt consumption (g)	12.83 ± 3.6	10.8 ± 3.5	−2.03	<0.001	12.7 ± 3.3	13.06 ± 3.80	0.36	0.4

^*∗*^Difference between before and after the intervention.

## Data Availability

Data are available upon written request to the corresponding author.

## Abbreviations

abrHBM: Health belief model
CHWs: Community health workers.
